# Heterogeneity in *Mycobacterium tuberculosis* immunoreactivity in young children in Blantyre, Malawi: A community-based survey

**DOI:** 10.1371/journal.pgph.0007269

**Published:** 2026-10-09

**Authors:** Hannah M. Rickman, Mphatso D. Phiri, Hannah Mbale, Helena R. A. Feasey, Marriott Nliwasa, George Chagaluka, James A. Seddon, Henry C. Mwandumba, Katherine C. Horton, Marc Y. R. Henrion, Tisungane Mwenyenkulu, Kuzani N. Mbendera, Emily S. Nightingale, Elizabeth L. Corbett, Peter MacPherson

**Affiliations:** 1 Clinical Research Department, London School of Hygiene & Tropical Medicine, London, United Kingdom; 2 Malawi Liverpool Wellcome Research Programme, Blantyre, Malawi; 3 Department of Clinical Sciences, Liverpool School of Tropical Medicine, Liverpool, United Kingdom; 4 Helse Nord TB Initiative, Kamuzu University of Health Sciences, Blantyre, Malawi; 5 School of Medicine, University of St Andrews, St Andrews, United Kingdom; 6 Department of Paediatrics, Queen Elizabeth Central Hospital, Blantyre, Malawi; 7 Department of Infectious Disease, Imperial College London, London, United Kingdom; 8 Desmond Tutu TB Centre, Department of Paediatrics and Child Health, Stellenbosch University, Stellenbosch, South Africa; 9 Department of Infectious Disease Epidemiology, London School of Hygiene & Tropical Medicine, London, United Kingdom; 10 Malawi National Tuberculosis and Leprosy Elimination Programme, Lilongwe, Malawi; 11 School of Health and Wellbeing, University of Glasgow, Glasgow, United Kingdom; Boston University, UNITED STATES OF AMERICA

## Abstract

As tuberculosis (TB) incidence declines, transmission becomes increasingly concentrated in vulnerable populations and neighbourhoods. There is an urgent need for affordable surveillance strategies to monitor trends, identify high-risk groups and target interventions. TB disease prevalence surveys are no longer cost-effective in monitoring epidemiological trends as countries target TB elimination, while passive surveillance methodologies such as case-notification rates (CNRs) are biased by healthcare access disparities. *Mycobacterium tuberculosis* (Mtb) immunoreactivity surveys indirectly measure transmission and therefore undiagnosed infectious TB, and may provide valuable information to TB programmes globally. We conducted a pragmatic hybrid clinic- and household-based cross-sectional interferon-gamma release assay (IGRA) survey in healthy children aged 1–4 years in 33 high-burden neighbourhoods Blantyre, Malawi. Participants were recruited using novel convenience sampling in health facilities, alongside random household sampling guided by satellite imagery, and tested for Mtb immunoreactivity using QFT-Plus IGRA. We constructed hierarchical Bayesian logistic regression models for IGRA positivity, with neighbourhood-level random effects. Of 1,545 participants, 102 (6.6%) had a positive IGRA: an annual risk of Mtb infection (ARTI) of 2.7% (95% CrI 2.2-3.2%). Immunoreactivity was higher in the poorest third of households (8.7% vs 4.9%; adjusted odds ratio: 1.88, 95% CrI 1.08-3.01) compared to the richest, but was not associated with HIV exposure, malnutrition or reported household TB exposure. There was substantial between-neighbourhood heterogeneity (ARTI range 1.1-4.1%). There was no association between neighbourhood-level TB case notifications and ARTI. Measuring Mtb immunoreactivity in young children, using innovative recruitment strategies, revealed a high burden of immunoreactivity suggestive of recent TB transmission. Children in the poorest households were at highest risk, highlighting the importance of social and economic inequity in driving the TB epidemic. Case notification rates may not reflect the areas with the highest transmission, and Mtb immunoreactivity surveillance may support identification of high-risk populations, monitoring of trends and targeting of public health interventions.

## Introduction

Tuberculosis (TB) remains the greatest global cause of mortality from any single infectious disease, killing an estimated 1.23 million people in 2024 [1]. As global prevalence falls, TB transmission, maintained by undiagnosed disease, becomes increasingly concentrated into vulnerable populations and geographies [2,3], and public health interventions need increasingly efficient targeting to avoid low yield and high costs [4]*.* Surveillance based on *Mycobacterium tuberculosis* (Mtb) immunoreactivity, indicating previous exposure and infection with Mtb, was widely used by countries with declining TB epidemics in the past century, and may add valuable information to current surveillance approaches [5].

Unlike for many other pathogens, antibodies are not a reliable indicator of Mtb exposure, with immunosurveillance instead requiring evidence of T-cell reactivity using tuberculin skin tests (TSTs), interferon-gamma release assays (IGRAs) or novel skin tests. Mtb immunoreactivity in young children, by definition, provides evidence of relatively *recent* infection (within their lifetimes), implying ongoing Mtb transmission from untreated infectious TB in the community [5]. Young children are at high risk of progression to severe disease and at lower risk of side effects from TB preventive treatment (TPT), and may therefore be more likely to benefit from TPT than other groups [6,7].

Mtb immunoreactivity surveys offer complementarity to disease surveillance methodologies, such as programmatic monitoring of case notification rates (CNRs) or TB disease prevalence surveys. CNRs only capture individuals diagnosed with TB through healthcare systems, thereby excluding the crucial “missing millions” – the estimated quarter of people with TB who go undiagnosed annually [1]. The World Health Organization (WHO) has previously recommended TB disease prevalence surveys in high-prevalence countries with likely low case-detection rates [8], but these require extremely large sample sizes, are usually only powered for national-level precision, and are prohibitively expensive in constrained healthcare systems [5].

Malawi, in Southern Africa, has reported remarkable progress towards TB elimination [1,9,10]. Blantyre, Malawi’s second largest city, is a densely-populated, resource-limited urban setting with an estimated adult HIV prevalence of 15% [11], which exemplifies many of the TB surveillance challenges discussed above. Blantyre’s TB prevalence has declined substantially from 1,014 per 100,000 adults in 2014–215 per 100,000 in 2019–2020, likely driven by strengthened decentralised TB services, successful HIV care scale-up, and intensive community-wide TB case-finding [9]. TB burden is unevenly distributed, with genomic analysis suggesting geographic areas of higher transmission [12]. TB CNRs vary significantly across Blantyre [13], but show evidence of an “inverse care law”: notifications are lower in poorer communities with worse healthcare access [14], suggesting that prioritising apparently “high-burden” areas with high CNRs may in fact divert resources away from areas of underdiagnosis. In this epidemiological context, cross-sectional disease prevalence surveys are very expensive and may offer limited additional insights: a 2019–2020 TB disease survey enrolled over 15,000 adult Blantyre residents and diagnosed just 29 individuals with bacteriologically-confirmed TB [9], generating limited power to identify high-burden areas [13].

As TB epidemics decline, efficient new approaches are required to reliably identify areas of higher recent TB transmission and guide targeted interventions. In a time of critical funding shortages, it is essential that these are pragmatic and affordable. We therefore performed a Mtb immunoreactivity survey utilising an innovative clinic-based convenience-sampling approach in children aged 1–4 years in high-risk areas of Blantyre, to determine the annual risk of Mtb infection and identify high-risk individuals and neighbourhoods.

## Methods

Detailed methods are in the published study protocol [15]. Input into the study design and materials was provided by the Malawi Liverpool Wellcome Programme TB/HIV Community Scientific Advisory Board, comprising community-members impacted by TB and/or HIV. The study is reported in accordance with the Strengthening the Reporting of Observational Studies in Epidemiology (STROBE) guidelines (S1 Checklist).

### Ethical statement

Ethical approval was received from the Kamuzu University of Health Sciences (Ref P.04/22/3611) and the London School of Hygiene and Tropical Medicine (Ref 2774).

### Setting

Participants were recruited 4 November 2022-24 March 2024 from 33 informal urban neighbourhoods of Blantyre, Malawi. Neighbourhoods were based on Community Health Worker catchment areas, encompassing the approximate catchment areas of the three primary health clinics (PHCs) in Blantyre with the highest number of TB case notifications [15].

### Participants & recruitment

Recruitment followed two approaches: clinic-based convenience sampling in the three PHCs, and random household sampling in the study neighbourhoods. We included children aged 1–4 years (12–60 months) normally resident in the study neighbourhoods. There were few exclusion criteria (see below), aiming to recruit a community-representative sample.

### Clinic convenience sample

In the clinic convenience sampling approach, eligible children were identified from those accessing or accompanying others to routine primary health services (e.g., vaccination clinics, check-ups, antenatal care, family planning). Children attending for their own symptomatic illness, or for TB/HIV care, were ineligible. Caregivers waiting for services were approached sequentially until daily recruitment targets were met.

### Random household sampling

Enumeration of possible household structures was performed using an open-access dataset of building footprints from OpenBuildings [16]. Population-weighted random samples were drawn in each neighbourhood, and selected locations visited by study teams, with all age-eligible children in the household approached for recruitment.

### Sample size

Sample sizes were based on precisely estimating an immunoreactivity prevalence of 5% with absolute precision of ±1% and 95% confidence, requiring 1,825 children 1–4 years with a QFT-Plus result, or 100 children with a positive result.

### Informed consent

Caregivers of eligible children were provided with oral and written information about the study and invited to provide individual informed consent in writing or, if illiterate, by a thumbprint observed and countersigned by an independent witness.

### Laboratory methods

A 4mL venous blood sample was taken for IGRA testing using QuantiFERON-TB Gold Plus (QFT-Plus, Qiagen).

### Results & follow-up

Where possible, we repeated QFT-Plus for participants with indeterminate results. Participants with a positive IGRA were clinically reviewed by a member of the study team. Participants with any symptoms were referred to the tertiary paediatric TB clinic for medical assessment and investigation of presumptive TB. Asymptomatic children with a positive IGRA were referred to local TB clinics with a recommendation to initiate TPT following Malawi National Guidelines. Their household contacts were offered TB disease screening.

### Covariables

Covariables including HIV status, HIV exposure (defined as positive maternal HIV status, regardless of infant status), Bacillus Calmette-Guérin (BCG) vaccination, previous TB treatment and TB household exposure were ascertained through caregivers’ report and patient-held health records. Wealth status was determined using a proxy means test developed from the Malawi Integrated Household Survey [17,18]. Mid-upper-arm circumference (MUAC) was measured and an age- and sex-adjusted z-score calculated, with malnutrition defined as a z-score ≤-2. Home locations were captured using direct GPS capture for household recruits; for clinic participants, a validated electronic participant location (ePal) app was used to assist participants to geolocate their homes using satellite images and local landmarks [14].

Neighbourhood-level poverty estimates were extracted from a 2019–2020 TB/HIV prevalence survey [9]. Neighbourhood-level HIV prevalence estimates were previously generated using spatially-explicit Bayesian regression from various data sources [11]. Enhanced surveillance of TB case notifications has been performed in Blantyre since 2011; neighbourhood-level CNRs were calculated for January 2021-June 2023 [14]. Denominators used 2020 unconstrained gridded population estimates from WorldPop, which were also used to calculate neighbourhood population density [19].

The outcome was a positive QFT-Plus IGRA; indeterminate results were excluded from prevalence calculations. We estimated annual risk of Mtb/TB infection (referred to with the commonly-used acronym ARTI) using the formula ARTI = 1-(1-prevalence)^1/mean age^ and calculated 95% credible intervals by jointly modelling prevalence and mean age as probabilistic quantities using a beta posterior distribution and normal distribution respectively, then drawing 10,000 samples from the joint posterior distributions and calculating the corresponding infection risk to develop a posterior distribution of ARTI estimates. As the estimated risk difference between clinic and household recruits was negligible, we pooled these groups for subsequent analyses.

### Data analysis

Analyses used R version 4·2 and the ‘brms’ interface to Stan. We constructed hierarchical Bayesian logistic regression models for an outcome of positive IGRA, with neighbourhood-level random effects, and calculated odds ratios by summarising 8000 posterior draws. We then explored the addition of individual- and neighbourhood-level covariables to the model, based on i) evidence of effect from univariable analyses and ii) *a priori* knowledge of TB transmission predictors. Models were fit with weakly-informative priors and 4 chains with 4000 iterations. Model convergence was checked by inspection of trace plots, effective sample sizes and $\hat{\text{R}}$ values, with posterior predictive checks used to assess model fit. We assessed collinearity by inspecting a correlation matrix and measuring variance inflation factors, including only non-colinear variables.

We constructed a first model for absolute Mtb immunoreactivity risk associated with residence in a specific neighbourhood, by adjusting for age and sex only and summarising neighbourhood-level random effects (expressed as odds ratios [ORs]). We then added additional individual- and neighbourhood-level covariables to measure their association with Mtb immunoreactivity, and subsequently constructed a second adjusted model, incorporating individual- and neighbourhood-level covariables identified as associated with the outcome, summarising neighbourhood-level random effects again to understand the extent of residual neighbourhood-level variation unaccounted for by the given covariables. Moran’s I was used to assess for autocorrelation in model residuals, using an adjacency matrix based on shared neighbourhood boundaries. Sensitivity analyses were performed which adjusted for recruitment location, comparing these models with base models with respect to 1) covariables associated with risk of Mtb immunoreactivity, and 2) the ranking of neighbourhood-level random effects using a Spearman rank coefficient.

We predicted neighbourhood-level ARTI from the posterior distributions of the age- and sex-adjusted model. We hypothesised a scenario in which a TB programme targeted the highest-risk quartile of neighbourhoods, and therefore identified eight “priority” neighbourhoods with the highest modelled ARTI, and compared those to the neighbourhoods with the highest CNRs.

The code to reproduce analyses is available at https://datacompass.lshtm.ac.uk/id/eprint/5362/ along with a summary dataset (home location has been removed to preserve confidentiality).

## Results

### Participant characteristics

We screened 2,882 children, of whom 2,581 (90%) were eligible for inclusion; 1,851 (72%) were consented for enrolment (S1 Fig). IGRA results were available for 1,735 (94%) participants, of which 190 (11%) were indeterminate and excluded from this analysis. In total, we included results from 1,545 participants, with a median age of 2.3 (interquartile range [IQR] 1.5-3.5) years (Table 1). 754 (49%) were female. 297 (19%) had been exposed to maternal HIV, of whom 17 (1.1% overall) were HIV positive. Almost all (1,516, 98%) had received BCG vaccination, and three (0.2%) were reported to have been previously treated for TB. 56 (3.6%) were reported to have had a TB household exposure.

**Table 1:** Participant characteristics, by recruitment location.

Characteristic	Overall N = 1,545	Clinic N = 1,134	Household N = 411
*Individual characteristics*			
Age, years – median (IQR)	2.31 (1.50, 3.50)	2.02 (1.36, 3.18)	3.14 (2.09, 4.00)
Female	754/1545 (49%)	549/1134 (48%)	205/411 (50%)
HIV positive	17/1545 (1.1%)	14/1134 (1.2%)	3/411 (0.7%)
HIV exposure	297/1544 (19%)	238/1134 (21%)	59/410 (14%)
Current cough	253/1545 (16%)	191/1134 (17%)	62/411 (15%)
Any current TB symptoms	374/1545 (24%)	293/1134 (26%)	81/411 (20%)
Previous BCG vaccination	1,516/1540 (98%)	1,119/1131 (99%)	397/409 (97%)
Previous TB treatment	3/1543 (0.2%)	1/1134 (<0.1%)	2/409 (0.5%)
Caregiver-reported general health			
Excellent	461/1545 (30%)	341/1134 (30%)	120/411 (29%)
Good	953/1545 (62%)	695/1134 (61%)	258/411 (63%)
Fair	115/1545 (7.4%)	85/1134 (7.5%)	30/411 (7.3%)
Poor	16/1545 (1.0%)	13/1134 (1.1%)	3/411 (0.7%)
MUAC-Z below -2SD	45/1545 (2.9%)	29/1134 (2.6%)	16/411 (3.9%)
Hospital admission in lifetime	220/1545 (14%)	180/1134 (16%)	40/411 (9.7%)
Current school attendance	418/1545 (27%)	254/1134 (22%)	164/411 (40%)
Ever travelled outside of Blantyre	747/1545 (48%)	537/1134 (47%)	210/411 (51%)
*Household characteristics*			
Number of household members – median (IQR)	5.0 (4.0, 6.0)	4.0 (3.0, 6.0)	6.0 (4.0, 7.0)
Means score			
Poorest tertile	515/1545 (33%)	397/1134 (35%)	118/411 (29%)
Middle tertile	515/1545 (33%)	415/1134 (37%)	100/411 (24%)
Richest tertile	515/1545 (33%)	322/1134 (28%)	193/411 (47%)
Educational level of household head			
Primary or below	507/1545 (33%)	364/1134 (32%)	143/411 (35%)
Some secondary education	377/1545 (24%)	300/1134 (26%)	77/411 (19%)
Completed secondary or above	661/1545 (43%)	470/1134 (41%)	191/411 (46%)
Distance to clinic (km)^1^ – median (IQR)	1.30 (0.77, 2.13)	1.31 (0.77, 2.09)	1.27 (0.77, 2.18)
TB household contact	56/1545 (3.6%)	24/1134 (2.1%)	32/411 (7.8%)
*Outcome*			
IGRA result			
Negative	1,443/1545 (93%)	1,064/1134 (94%)	379/411 (92%)
Positive	102/1545 (6.6%)	70/1134 (6.2%)	32/411 (7.8%)
ARTI, % - estimate, 95% CrI	2.7 (2.2, 3.2)	2.7 (2.1, 3.4)	2.7 (1.8, 3.7)

TB: tuberculosis. BCG: Bacillus Calmette-Guérin. MUAC-Z: age- and sex-adjusted mid-upper arm circumference Z-score. SD: standard deviations. IQR: interquartile range. ARTI: annual risk of Mtb infection. CI: credible interval.

^1^Euclidean distance of home location to nearest primary health clinic.

Categorical variables are reported as number and percentage. Continuous variables are reported as a median and interquartile range, with the exception of ARTI, which is reported as an overall percentage estimate with 95% credible estimates calculated from the joint posterior distribution of prevalence and mean age, as described in the methods.

### Participants by recruitment site

Reasons for clinic attendance are reported in S1 Table.Participants recruited using clinic convenience sampling and random household sampling were similar with respect to sex, HIV status, current cough, caregiver-reported general health, previous TB, and nutritional status (Table 1). However, children recruited from clinics were younger, with a higher prevalence of HIV exposure and current symptoms, and from poorer households. Children recruited from households were more likely to have reported TB household exposure. These differences persisted when adjusted for age (S2 Table). Nevertheless, there was no evidence of difference in ARTI between clinic and household groups, with a predicted ARTI of 2.7% in both (estimated absolute risk difference 0.14%, 95% CrI -1.1 to 1.1%).

### IGRA positivity and ARTI

Overall, 102 (6.6%) participants had a positive IGRA, equating to an ARTI of 2.7% (95% CrI 2.2-3.2%) per year. The estimated prevalence was slightly higher in girls than in boys (7.6% vs 5.7%; adjusted OR [AOR] 1.38, 95%CI: 0.93-2.06) (Table 2). We did not find strong evidence for association between Mtb immunoreactivity and HIV exposure, malnutrition, distance to clinic or caregiver-reported TB household exposure, nor with neighbourhood characteristics such as neighbourhood-level poverty, HIV prevalence or population density. Children living in the poorest third of households were more likely to have Mtb immunoreactivity than the richest tertile (8.7% vs 4.9%; AOR 1.88, 95%CI: 1.08-3.01). There was no difference in associations when analyses were adjusted for recruitment location (clinic vs household) (S3 Table).

**Table 2:** Risk factors for Mycobacterium tuberculosis immunoreactivity (combined clinic convenience and random household participants). Odds ratios are expressed relative to the reference categories (for categorical variables), or per 1-unit increase.

Characteristic	IGRA Negative n = 1,443	IGRA Positive n = 102	OR^1^ (95% CrI)	AOR^2^ (95% CrI)
*Individual variables*				
Age (years)	2.32 (1.48, 3.50)	2.28 (1.70, 3.50)	1.03 (0.87-1.22)	1.03 (0.86-1.22)
Sex				
Male	746 (52%)	45 (44%)	*REF*	*REF*
Female	697 (48%)	57 (56%)	1.38 (0.93-2.07)	1.38 (0.93-2.06)
HIV exposure				
HIV unexposed	1,164 (81%)	83/101^3^ (82%)	*REF*	*REF*
HIV exposed	279 (19%)	18/101^3^ (18%)	0.88 (0.49-1.49)	0.87 (0.51-1.43)
MUAC				
MUAC-Z ≥ -2	1399 (97%)	101 (99%)	*REF*	*REF*
MUAC-Z below -2	44 (3%)	1 (1%)	0.29 (0.03-1.5)	0.28 (0.03-1.43)
*Household variables*				
Household wealth				
Richest tertile	490 (34%)	25 (25%)	*REF*	*REF*
Middle tertile	483 (33%)	32 (31%)	1.29 (0.71-2.14)	1.34 (0.74-2.21)
Poorest tertile	470 (33%)	45 (44%)	1.87 (1.10-3.03)	1.88 (1.08-3.01)
Education level of household head				
Some secondary education	978 (68%)	60 (59%)	*REF*	*REF*
Primary education or below	465 (32%)	42 (41%)	1.37 (0.91-2.04)	1.39 (0.91-2.08)
Distance to clinic (km)	1.30 (0.77, 2.12)	1.32 (0.81, 2.25)	0.98 (0.72-1.36)	0.98 (0.72-1.33)
TB household exposure				
No TB household exposure	1,391 (96%)	98 (96%)	*REF*	*REF*
TB household exposure	52 (4%)	4 (4%)	1.05 (0.31-2.97)	1.04 (0.32-2.87)
*Neighbourhood variables*				
Neighbourhood adult HIV prevalence, %^4^	17.2 (16.6, 18.9)	17.7 (16.6, 20.2)	1.12 (0.96-1.30)	1.12 (0.95-1.30)
Median poverty score^5^	-5.76 (-6.67, -5.11)	-6.05 (-6.77, -5.23)	0.83 (0.64-1.07)	0.82 (0.64-1.07)
Population density, people per 100m^2^	1.79 (100, 347)	1.54 (99, 209)	0.81 (0.66-1.01)	0.81 (0.65-1.00)
TB case notification rate, cases per 1,000 per year^6^	1.53 (1.16, 1.85)	1.53 (1.16, 1.85)	1.17 (0.70-2.08)	1.17 (0.70-2.03)

IGRA: Interferon-gamma release assay. OR: Odds ratio. AOR: Adjusted odds ratio. CrI: Credible interval. MUAC-Z: Age- and sex-adjusted mid-upper arm circumference Z-score. TB: Tuberculosis.

^1^Odds ratios from a multilevel Bayesian logistic regression model, with random intercepts for neighbourhood. ^2^As above, but additionally adjusted for age and sex. ^3^Data missing for one participant. ^4^See methods. ^5^See methods; higher (i.e., less negative) scores represent greater poverty. ^6^CNRs scaled to cases per 1,000 per year (to aid interpretability of odds ratios).

### Neighbourhood-level variability

There was substantial between-neighbourhood heterogeneity in Mtb immunoreactivity prevalence in the age- and sex-adjusted hierarchical model, (S1 Text), accounting for around 9% of total variability (intraclass correlation coefficient 0.087, 95% CrI 0.017-0.21). High prevalence of Mtb immunoreactivity was observed in several peri-urban neighbourhoods around Bangwe health centre and in some neighbourhoods around Ndirande (Fig 1A). Predicted neighbourhood ARTI ranged from 1.11% (0.32-2.42%) to 4.11% (1.70-8.30%). Participants in the highest-risk neighbourhood had twice the odds of Mtb immunoreactivity of the overall cohort (OR 1.98, 95% CrI: 0.86-4.57), while those in the lowest-risk neighbourhood had half the odds (OR 0.48, 95% CrI: 0.20-1. 02) (S2 Fig). In sensitivity analyses which adjusted for recruitment location, there was little change in the ranking of the neighbourhoods by risk (Spearman coefficient 0.998).

**Fig 1:**
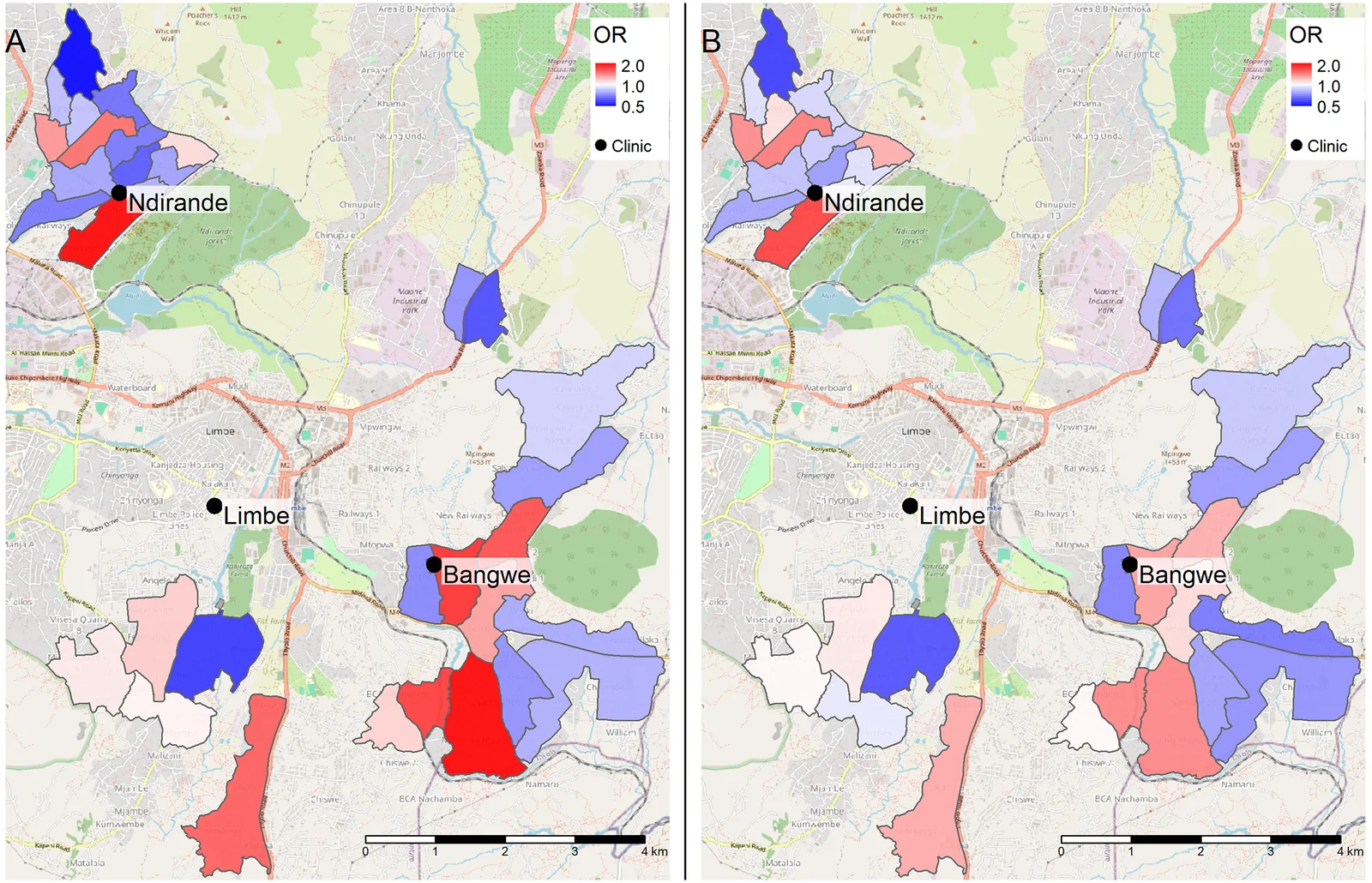
Odds of Mtb immunoreactivity associated with residence in each neighbourhood: neighbourhood-level Mtb immunoreactivity random effects, expressed as odds ratios. **A.** Age- and sex-adjusted model, showing absolute odds ratios associated with neighbourhoods. **B.** Model including age, sex, household poverty, neighbourhood HIV prevalence, and population density, showing residual neighbourhood-specific random effects once risk factors are accounted for. Map tile data from OpenStreetMap (https://www.openstreetmap.org/copyright). Neighbourhood shapefiles were produced based on community health worker catchment areas for a cluster-randomised trial and are freely available at https://github/petermacp/mlwdata OR: odds ratios.

Many potentially-relevant covariables also varied spatially (S3 Fig); we therefore constructed an extended model which included age, sex, household poverty, and neighbourhood-level HIV prevalence and population density (S1 Text). There was some evidence for higher Mtb immunoreactivity in areas with high HIV prevalence (AOR 1.08 per 1% increase in HIV prevalence; 95% CrI 0.93-1.25)) and with lower population density (AOR 0.84 per person per 100m^2^ increase, 95% CrI 0.67-1.04). Adding covariables did not substantially improve model fit (S1 Text), and resulted in a modest reduction in between-neighbourhood variability (ICC 0.055, 95% CrI 0.001-0.179) and the magnitude of the remaining neighbourhood-level random effects (Fig 1B). There was no evidence of residual spatial autocorrelation in either model using Moran’s I (S1 Text).

### Comparison with case notifications

There was no association between areas with high TB CNRs, and high risk of Mtb immunoreactivity (AOR 1.17 per 1 per 1,000 increase in CNR, 95% CrI 0.70-2.03; Fig 2). Of eight hypothetical “priority” neighbourhoods identified as having the highest ARTI, only two were also within the eight areas with the highest CNR.

**Fig 2:**
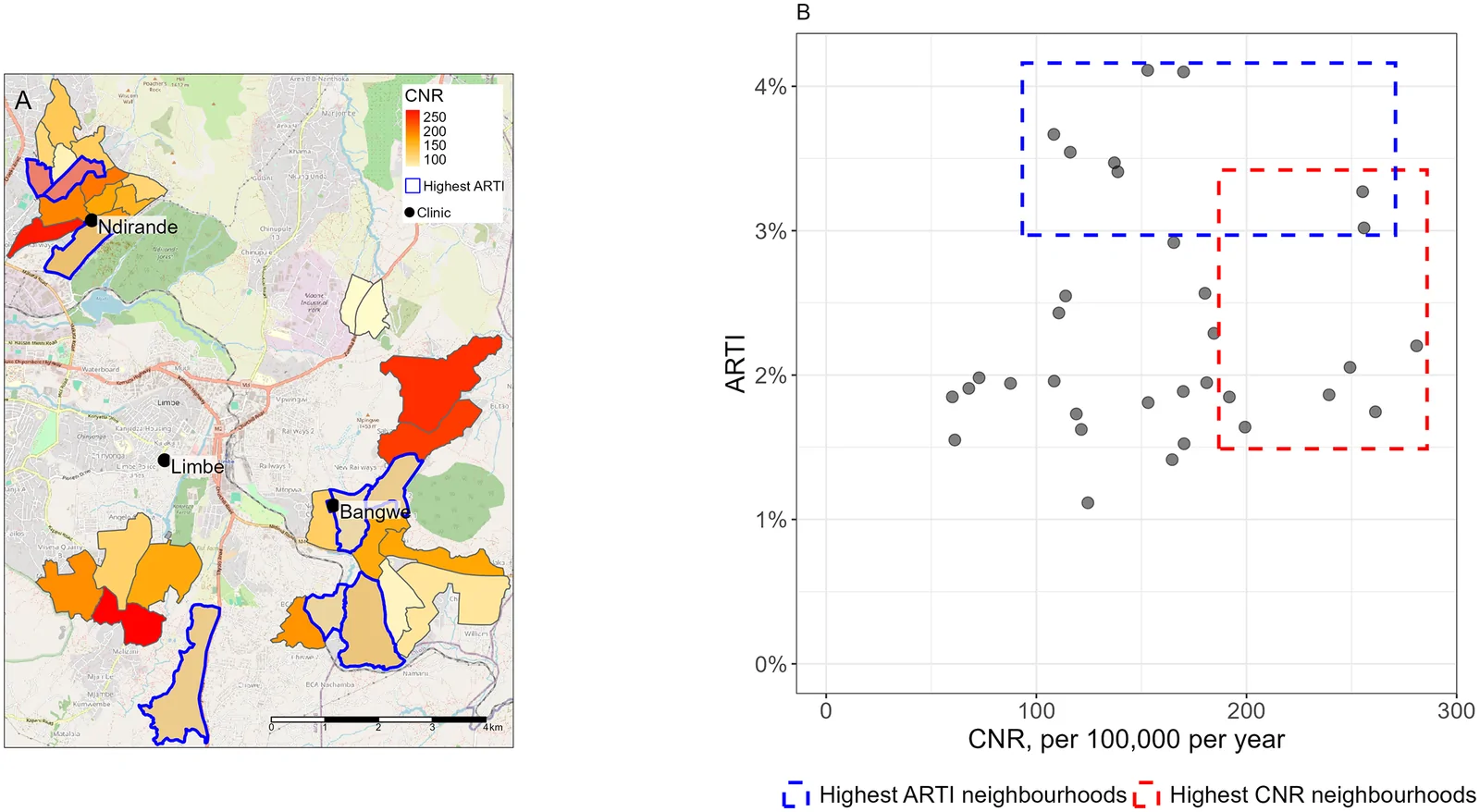
Correlation between neighbourhood-level case notification rates (CNR) and annual risk of Mtb infection (ARTI). Map tile data from OpenStreetMap (https://www.openstreetmap.org/copyright). Neighbourhood shapefiles were produced based on community health worker catchment areas for a cluster-randomised trial and are freely available at https://github/petermacp/mlwdata. **A.** Case notification rates per 100,000 per year, by neighbourhood. Areas highlighted in blue are the eight neighbourhoods with the highest ARTI, which might theoretically be targeted for intervention by a TB programme if they were to prioritise based on ARTI, rather than CNR. **B.** Neighbourhood-level correlation between CNR and ARTI. Again, the neighbourhoods which would be targeted for intervention based on ARTI are highlighted in blue, while those which would be targeted for intervention based on CNR are highlighted in red.

## Discussion

We used innovative convenience- and random household-sampling methodologies to conduct an Mtb immunoreactivity survey in a city with a rapidly evolving TB epidemic, and found evidence of a substantial (2.7%) annual risk of TB transmission to young children. Mtb immunoreactivity was higher in children from the poorest households and in some peri-urban neighbourhoods, indicating local heterogeneity in TB epidemiology. Our findings suggest that Mtb immunoreactivity surveillance may allow modelling of local TB transmission trends and identification of high-risk neighbourhoods for public health interventions, as well as identifying TB-exposed children who may benefit from TPT.

Global health funding cuts demand that any surveillance methodology optimises cost-effectiveness and efficiency, for example by integrating within existing systems such as primary care. Our clinic-based approach built on a strategy of opportunistically screening clinic attendees for malaria parasitaemia in order to identify high-transmission villages in Chikwawa, Malawi [20]. We did note some differences in the characteristics of participants recruited via clinic-based convenience versus cross-sectional random household sampling. These differences may reflect information bias (data was gathered on household recruits within their own household, and typically from the head of household, which may have affected responses to questions on poverty and maternal HIV status) or true selection differences (children attending clinic for vaccinations or accompanying family members were on average younger), or a combination. Nevertheless, the overall ARTI estimates were very similar, suggesting that in this context clinic-based convenience sampling could offer a cost-effective alternative to random household sampling. Clinic-based surveillance may facilitate linkage to care for participants eligible for TPT and offers opportunities to integrate within multi-disease surveillance systems.

The main individual risk factor for Mtb immunoreactivity was household poverty, with children in the poorest third of households having 1.9-fold higher odds of IGRA positivity compared to the richest tertile. There are multiple interacting mechanisms by which household poverty can increase TB risk, including: crowding, housing conditions, exposure to congregate spaces, nutrition, air quality, healthcare access, and co-association with factors such as HIV and smoking. The relationship between poverty and TB is complex and often context-specific [21,22], but our findings support poverty alleviation and social protection for vulnerable communities as essential components of global efforts to end TB.

We found no association between maternal HIV status and child Mtb immunoreactivity; previous research is mixed [23–25]. Similarly, while malnutrition is strongly associated with TB *disease*, we did not observe an association with Mtb immunoreactivity, in line with the theory that malnutrition may predominantly impact progression from infection to disease [26].

The lack of association between TB household exposure and Mtb immunoreactivity may partially reflect challenges in accurately ascertaining TB exposure, and is based on only a small number of positive children. Nevertheless, this finding is compatible with knowledge that most TB transmission to young children occurs outside the household [27], and that while young children’s TB exposure may notbe fully representative of community transmission, it is likely to be impacted by their wider local environment. Indeed, as young children are typically less mobile within cities than adolescents and adults, Mtb immunoreactivity in this age group may serve as a powerful sentinel of undiagnosed infectious TB in their neighbourhood.

We found a four-fold variation in estimated ARTI by neighbourhood, indicating possible priority areas for public health action. This is despite the study being conducted within a single, relatively homogeneous city, in purposively selected informal neighbourhoods with higher TB CNRs; transmission is likely even more heterogeneous in more unequal settings. Modelling work suggests that spatial targeting of TB interventions such as active case-finding may disproportionately impact transmission [28]; however very few studies describe *implementation* of spatially-targeted TB interventions in high-incidence settings, in part due to the challenges in identifying high-risk neighbourhoods and in evaluating the impact of such interventions [2,29]. Spatially-concentrated transmission may arise due to a combination of environmental factors such as crowding, distribution of population factors such as HIV prevalence, and local “outbreaks” of transmission [12]. In this study, known risk factors such as HIV prevalence and population density accounted for only a minority of the neighbourhood-level variation in risk, suggesting an additional contribution from local transmission. Furthermore, these relationships were complex. For example, we found weak evidence of a *negative* association between population density and Mtb immunoreactivity: plausible in the Blantyre context, as neighbourhoods around one clinic (Ndirande) are dense but well-established, with relatively good access to public healthcare, whereas lower-density peri-urban regions around a second clinic (Bangwe) represent more recent informal urban expansion. This reinforces the complexities in predicting local TB risk and the need for context-specific epidemiological data.

Importantly from a programmatic perspective, CNRs did not predict Mtb immunoreactivity, highlighting the limitations of CNRs as indicators of undiagnosed infectious disease within communities; indeed, higher CNRs may simply reflect greater access to healthcare [14]. Several TB programmes are targeting high-CNR areas for enhanced intervention, but in this setting only two of the “priority” highest-risk quartile of neighbourhoods based on CNR were in the highest quartile of risk measured by ARTI. Combining Mtb immunoreactivity measurements with disease prevalence surveys (in specific settings), genomic sequencing, markers of TB severity, or predictors of underdiagnosis, may add further triangulation and power to understand which areas truly have the highest burden of undiagnosed disease [5,12,13]. Further value in Mtb immunoreactivity surveillance may come in measuring trends rather than in one-off surveys, and future surveys may add further data to this picture.

The 2.7% ARTI was higher than historically observed in limited previous data from Malawi. The 1994 Malawi National Tuberculin survey in 6–11-year-olds estimated an ARTI of 1% (varying from 0.7%-2.2% by region) [30], while a 2012 TST survey in 2–4-year-olds in rural Northern Malawi, which used a strict 15mm cut-off, found an ARTI of 0.3% [24]. Blantyre has a high TB incidence relative to the rest of Malawi, and the ARTI in our study may have been inflated by purposive selection of urban areas with high case-notifications. Additionally the children recruited lived through the COVID-19 pandemic, which may have caused delays in TB diagnosis and increased community transmission [31]. Furthermore, surveys in older age-groups which estimate ARTI without accounting for reversion may significantly under-estimate true transmission [5,32].

This study has several limitations. Firstly, most participants were recruited through a primary care-based convenience sampling approach. This was partly justified by the high uptake of primary healthcare in Malawi, and the logistical and cost advantages; it is noteworthy that conventional household-based sampling methods are also subject to selection biases. Our approach is supported by similar results from clinic and household-based groups; however, we did note some differences in the characteristics of these children which may suggest the clinic-based group may not have been fully representative. From a statistical perspective, the non-probabilistic sampling approach limits formal inference to the wider population. The utility of convenience sampling is likely to vary between settings: Malawi has an established unified public primary healthcare system, but clinic-based sampling would be more problematic in places with multiple public and private providers, or with more uneven access to care. Broader application would require context-specific validation, such as the hybrid strategy employed here, which uses a smaller, random household-based sample to validate the more pragmatic clinic-based estimates. Integrating Mtb immunoreactivity testing into ongoing programmes such as Demographic Health Surveys or Multiple Indicator Cluster Surveys might improve comparability of results between settings. We observed an appreciable number of indeterminate results. Some of the covariables in our model are themselves estimates (for example HIV prevalence and Worldpop estimates); nevertheless, these reflect the available data sources for policy-makers in settings such as Blantyre.

More broadly, Mtb immunoreactivity prevalence, and calculated ARTI, have limitations as measures of community force of infection [5]. There is no “gold-standard” test for Mtb infection, and Mtb immunoreactivity does not fully capture exposure or individual risk. IGRA performance in young children appears comparable to older age groups [33], but there is less data from children under two years. Recruiting young children prioritises more recent transmission, but their immunoreactivity nevertheless reflects the past few years of cumulative exposure, which will correlate imperfectly with current transmission in rapidly-changing epidemics. Population mobility limits spatial inference, as infection may not have been acquired in the participant’s home neighbourhood. Classical calculations of ARTI do not take into account reversion, which may lead to significant underestimation of the true force of infection [32].

## Conclusion

TB remains a major global killer, and countries are not on track to achieve ambitious 2030 End TB goals [1]. Context-specific surveillance systems and timely data are required to design targeted strategies to address complex, heterogeneous and changing TB epidemiology. This study highlights the potential of IGRA surveillance using an innovative clinic-based convenience sampling as a tool for monitoring Mtb transmission, identifying high-risk areas, and targeting interventions to optimise use of increasingly limited resources. Mtb infection was associated with poverty, and it is critical to address the social determinants of TB to prevent morbidity and mortality, and to achieve TB elimination goals.

## Supporting information

S1 Checklist STROBE checklist. Reproduced from the STROBE Statement under a Creative Commons Attribution 4.0 International (CC BY 4.0) licence. Available from: https://www.strobe-statement.org/
(DOC)

S1 Fig Flow chart of participants screened and recruited into the study. (TIF)

S2 Fig Neighbourhood-level odds for Mtb immunoreactivity. Forest plot showing neighbourhood-level estimated odds ratios (ORs) for *Mycobacterium tuberculosis* (Mtb) immunoreactivity, relative to a reference neighbourhood with a mean random effect (i.e., OR = 1). Each density ridge represents the full posterior distribution of the neighbourhood-level random effect.
(TIFF)

S3 Fig Spatial variation of neighbourhood-level covariables. Neighbourhood shapefiles were produced based on community health worker catchment areas for a cluster-randomised trial and are freely available at https://github/petermacp/mlwdata. A. HIV prevalence amongst adults aged 15–49 years (data previously generated using spatially-explicit Bayesian regression from the 2019 survey and two further national HIV prevalence surveys [11]). B. Population density (people per 100m^2^, i.e., 10m x 10m), from unconstrained gridded population estimates for 2020 from Worldpop [19]. C. Median poverty score using a proxy means test; higher (i.e., less negative) scores indicate higher chance of poverty. Data from a 2019 TB prevalence survey [9].
(TIFF)

S1 Table Reasons for clinic attendance amongst participants recruited from clinic. (DOCX)

S2 Table Age-adjusted associations between covariables and recruitment location. (DOCX)

S3 Table Sensitivity analysis of models of risk factors for Mtb immunoreactivity adjusted for recruitment location in addition to age and sex. (DOCX)

S1 Text Simple and full models for risk of Mtb immunoreactivity. (DOCX)
