# Supplementary material for: Heterogeneity in Mycobacterium tuberculosis immunoreactivity in young children in Blantyre, Malawi: A community-based survey
Source: PLOS Glob Public Health. 2026 Oct 9;6(10):e0007269. doi: 10.1371/journal.pgph.0007269 (PMC13652165; doi:10.1371/journal.pgph.0007269)
Supplement: Supplementary file 1 [file pgph.0007269.s005.docx]

S1 table - Reasons for clinic attendance amongst participants recruited from clinic

| ***Reason for clinic attendance*** | **N (%)** |
| --- | --- |
| Accompanying another person attending family planning / cervical screening | 455 (40.1%) |
| For check-up / weighing | 401 (35.4%) |
| Accompanying another person attending antenatal / postnatal care | 86 (7.6%) |
| Accompanying another person attending for vaccinations | 66 (5.8%) |
| Accompanying another person attending outpatient department | 36 (3.2%) |
| For vaccinations | 25 (2.2%) |
| Other* | 65 (5.7%) |

*Note participants excluded if attending for their own illness, or if accompanying someone attending TB or HIV clinic
