# Supplementary material for: Heterogeneity in Mycobacterium tuberculosis immunoreactivity in young children in Blantyre, Malawi: A community-based survey
Source: PLOS Glob Public Health. 2026 Oct 9;6(10):e0007269. doi: 10.1371/journal.pgph.0007269 (PMC13652165; doi:10.1371/journal.pgph.0007269)
Supplement: Supplementary file 2 [file pgph.0007269.s006.docx]

S2 Table - Age-adjusted associations between covariables and recruitment location

| Characteristic | Clinic N = 1,134 | Household  N = 411 | OR (Household vs clinic) | AOR (Household vs clinic) |
| --- | --- | --- | --- | --- |
| Sex |  |  |  |  |
| Male | 585 (52%) | 206 (50%) | REF | REF |
| Female | 549 (48%) | 205 (50%) | 1.06 (0.85-1.32) | 1.07 (0.86-1.35) |
| HIV status |  |  |  |  |
| HIV negative | 1120 (99%) | 408 (99%) | REF | REF |
| HIV positive | 14 (1%) | 3 (1%) | 0.55 (0.15-1.70) | 0.42 (0.11-1.38) |
| HIV exposure |  |  |  |  |
| HIV unexposed | 896 (79%) | 351/410 (86%) | REF | REF |
| HIV exposed^1^ | 238 (21%) | 59/410 (14%) | 0.63 (0.47-0.86) | 0.57 (0.41-0.78) |
| Current cough |  |  |  |  |
| No cough | 943 (83%) | 349 (85%) | REF | REF |
| Cough | 191 (17%) | 62 (15%) | 0.87 (0.64-1.19) | 0.87 (0.62-1.21) |
| Any current TB symptoms |  |  |  |  |
| No symptoms | 841 (74%) | 330 (80%) | REF | REF |
| Symptoms | 293 (26%) | 81 (20%) | 0.70 (0.53-0.93) | 0.75 (0.56-0.98) |
| Previous TB treatment |  |  |  |  |
| Never treated for TB | 1133 (>99.9%) | 407/409 (99.5%) | REF | REF |
| Ever treated for TB^2^ | 1 (<0.1%) | 2/409 (0.5%) | 4.25 (0.44-46.6) | 5.82 (0.58-70.0) |
| Hospital admission |  |  |  |  |
| Never admitted | 954 (84%) | 371 (90%) | REF | REF |
| Ever admitted | 180 (16%) | 40 (10%) | 0.57 (0.39-0.81) | 0.55 (0.38-0.79) |
| School attendance |  |  |  |  |
| No | 880 (78%) | 247 (60%) | REF | REF |
| Yes | 254 (22%) | 164 (40%) | 2.29 (1.80-2.94) | 1.34 (1.01-1.76) |
| Travel outside of Blantyre |  |  |  |  |
| Never | 597 (53%) | 201 (49%) | REF | REF |
| Ever | 537 (47%) | 210 (51%) | 1.16 (0.93-1.45) | 1.12 (0.88-1.41) |
| Number of household members | 4.0 (3.0, 6.0) | 6.0 (4.0, 7.0) | 1.28 (1.22-1.35) | 1.26 (1.19-1.33) |
| Wealth tertile |  |  |  |  |
| Richest two tertiles | 737 (65%) | 293 (71%) | REF | REF |
| Poorest tertile | 397 (35%) | 118 (29%) | 0.75 (0.58-0.95) | 0.69 (0.53-0.88) |
| Household head education |  |  |  |  |
| Secondary or above | 770 (68%) | 268 (65%) | REF | REF |
| Primary or below | 364 (32%) | 143 (35%) | 1.12 (0.88-1.43) | 1.04 (0.81-1.33) |
| TB household contact |  |  |  |  |
| No | 1110 (98%) | 379 (92%) | REF | REF |
| Yes | 24 (2%) | 32 (8%) | 3.86 (2.19-6.83) | 3.70 (2.07-6.55) |

OR: Odds ratio. AOR: Age-adjusted odds ratio TB: Tuberculosis.^1^Data missing for one participant ^2^Data missing for two participants

Odds ratios and age-adjusted ratios for each characteristic in household, as compared to community, participants, +/- adjusted for age. Odds ratios are expressed relative to the reference categories (for categorical variables), or per 1-unit increase in continuous variables. Continuous variables are summarized as median (interquartile range).
