# Supplementary material for: Heterogeneity in Mycobacterium tuberculosis immunoreactivity in young children in Blantyre, Malawi: A community-based survey
Source: PLOS Glob Public Health. 2026 Oct 9;6(10):e0007269. doi: 10.1371/journal.pgph.0007269 (PMC13652165; doi:10.1371/journal.pgph.0007269)
Supplement: Supplementary file 3 [file pgph.0007269.s007.docx]

S3 table - Sensitivity analysis of models of risk factors for Mtb immunoreactivity adjusted for recruitment location in addition to age and sex

| Characteristic | OR^1^  (95% CrI) | AOR^2^  (95% CrI) | AOR from sensitivity analysis  (95% CrI) |
| --- | --- | --- | --- |
| Age (years) | 1.03  (0.87-1.22) | 1.03  (0.86-1.22) | 1.01  (0.84-1.2) |
| Sex |  |  |  |
| Male | *REF* | *REF* | *REF* |
| Female | 1.38  (0.93-2.07) | 1.38  (0.93-2.06) | 1.38  (0.91-2.08) |
| HIV exposure |  |  |  |
| HIV unexposed | *REF* | *REF* | *REF* |
| HIV exposed | 0.88  (0.49-1.49) | 0.87  (0.51-1.43) | 0.88  (0.50-1.48) |
| MUAC |  |  |  |
| MUAC-Z ≥ -2 | *REF* | *REF* | *REF* |
| MUAC-Z below -2 | 0.29  (0.03-1.5) | 0.28  (0.03-1.43) | 0.28  (0.03-1.52) |
| Household wealth |  |  |  |
| Richest tertile | *REF* | *REF* | *REF* |
| Middle tertile | 1.29  (0.71-2.14) | 1.34  (0.74-2.21) | 1.01  (0.69-1.47) |
| Poorest tertile | 1.87  (1.10-3.03) | 1.88  (1.08-3.01) | 1.61  (1.12-2.38) |
| Education level of household head |  |  |  |
| Some secondary education | *REF* | *REF* | *REF* |
| Primary education or below | 1.37  (0.91-2.04) | 1.39  (0.91-2.08) | 1.37  (0.91-2,13) |
| Distance to clinic (km) | 0.98  (0.72-1.36) | 0.98  (0.72-1.33) | 0.97  (0.71-1.32) |
| TB household exposure |  |  |  |
| No TB household exposure | *REF* | *REF* | *REF* |
| TB household exposure | 1.05  (0.31-2.97) | 1.04  (0.32-2.87) | 0.99  (0.31-2.57) |
| Neighbourhood adult HIV prevalence, %^4^ | 1.12  (0.96-1.30) | 1.12  (0.95-1.30) | 1.12  (0.97-1.31) |
| Median poverty score^5^ | 0.83  (0.64-1.07) | 0.82  (0.64-1.07) | 0.83  (0.64-1.06) |
| Population density, people per 100m^2^ | 0.81  (0.66-1.01) | 0.81  (0.65-1.00) | 0.81  (0.66-1.01) |
| TB case notification rate, cases per 1,000 per year^6^ | 1.17  (0.70-2.08) | 1.17  (0.70-2.03) | 1.18  (0.68-2.08) |
