# Supplementary material for: Heterogeneity in Mycobacterium tuberculosis immunoreactivity in young children in Blantyre, Malawi: A community-based survey
Source: PLOS Glob Public Health. 2026 Oct 9;6(10):e0007269. doi: 10.1371/journal.pgph.0007269 (PMC13652165; doi:10.1371/journal.pgph.0007269)
Supplement: Supplementary file 4 [file pgph.0007269.s008.docx]

S1 Text - Simple and full models for risk of Mtb immunoreactivity

Baseline model 1:

logit(Pr(QFTP_i_)) =	β_0_ + β_1_⋅Age_i_ + β_2_⋅Sex_i_ + u_neighbourhood[i]_

Extended model 2:

logit(Pr(QFTP_i_)) =	β_0_ + β_1_⋅Age_i_ + β_2_⋅Sex_i_ + β_3_⋅Poverty_i_ + β4⋅HIVPrev_neighbourhood[i]_ +

β5⋅Density_neighbourhood[i]_ + u_neighbourhood[i]_

Where:

QFTP_i_	Binary outcome for individual *i* (log-odds of QFTP positivity)

β_0_	Intercept (baseline log odds)

∼N(0, 5^2^)

β_1 ,_ β_2 ,_ β_3_	Fixed effects for individual covariables (age, sex, being in poorest tertile)

∼N(0, 2.5^2^)

β_4 ,_ β_5_	Fixed effects for neighbourhood covariables (HIV prevalence, population density)

∼N(0, 2.5^2^)

u_neighbourhood[i]_	Neighbourhood-level random intercept

∼N(0, σ^2^_neighbourhood_)

Model summary:

|  | Baseline model | Extended model |
| --- | --- | --- |
| Age *(odds ratio, 95% CrI)* | 1.03 (0.87-1.22) | 1.02 (0.85-1.20) |
| Sex (female) *(odds ratio, 95% CrI)* | 1.39 (0.92-2.09) | 1.36 (0.89-2.06) |
| Poorest third *(odds ratio, 95% CrI)* | NA | 1.88 (1.08-3.01) |
| HIV prevalence *(odds ratio, 95% CrI)* | NA | 1.08 (0.93-1.25) |
| Population density *(odds ratio, 95% CrI)* | NA | 0.84 (0.67-1.04) |
| Between-neighbourhood variability  *(Intercept sd)* | 1.76 (1.27-2.55) | 1.55 (1.06-2.33) |
| Intraclass correlation coefficient *(estimate, 95% CrI)* | 0.087 (0.017-0.21) | 0.055 (0.00-0.18) |
| LOOIC | 743.03 | 741.55 |
| WAIC | 742.88 | 741.39 |
| Moran’s I statistic *(estimate, p value)* | 0.03, p = 0.335 | 0.04, p = 0.309 |

sd: standard deviation. LOOIC: Leave-One-Out Information Criterion. WAIC: Widely-Applicable Information Criterion
